# Ex vivo live imaging of melanoblast migration in embryonic mouse skin

**DOI:** 10.1111/j.1755-148X.2010.00669.x

**Published:** 2010-04

**Authors:** Richard L Mort, Leonard Hay, Ian J Jackson

**Affiliations:** MRC Human Genetics Unit, Institute of Genetics and Molecular Medicine, Western General HospitalEdinburgh, UK

Dear Sir,

Melanoblasts are the embryonic precursors of melanocytes. They are derived from the neural crest at around embryonic day 9.5 (E9.5) and upregulate early melanoblast specific markers (*Mitf, Tyrosinase, Dct, Kit*) around E10.5. Subsequently, melanoblasts migrate along the dorsolateral pathway throughout the developing dermis (for a recent review see Thomas and Erickson, 2008). They are distributed apparently at random throughout the epidermis at E14.5 where they begin to localise to the developing hair follicles (Mayer, 1973). Little is known about the kinetics of melanoblast migration and localisation because of the difficulty in performing confocal imaging on live embryonic skin. Culture of embryonic skin is technically challenging because of the requirement for an air-liquid-interface (ALI) in order for the tissue to develop. Historically this has been achieved by culturing on floating polycarbonate membranes (Jordan and Jackson, 2000; Kashiwagi et al., 1997). Although this method has been used successfully for bright field imaging of developing cultures (Kashiwagi et al., 1997), it is not amenable to live imaging using confocal microscopy because the samples are not sufficiently immobilised and reflection artefacts created by the tissue surface make imaging problematic (data not shown).

In order to study melanoblast behaviour in the embryonic epidermis there was a requirement for: (a) a good melanoblast specific fluorescent reporter strain, (b) a method to immobilise the skin sample to prevent drift in the x, y and z dimensions, (c) a method to invert the sample so that it was compatible with a standard inverted microscope enclosure and (d) a method to maintain an ALI across the epidermal side of the tissue whilst also avoiding reflection artefacts. To achieve the first we labelled melanoblasts with yellow fluorescent protein (YFP) by combining *TyrCreB* mice, expressing *Cre* recombinase under the control of the *Tyrosinase* promoter (Delmas et al., 2003), with *R26YFPR* reporter animals that express YFP conditionally from the *ROSA26* locus (Srinivas et al., 2001). Figure 1 outlines the ex vivo embryonic skin culture system we have developed to address points b to d. Briefly, we sandwiched a freshly dissected E14.5 embryonic skin sample between a Nuclepore membrane (Whatman) and a gas permeable Lumox membrane (Greiner Bio-One GmbH) so that the epidermal side of the skin was in contact with the Lumox membrane. The whole assembly is mounted in a specially designed culture chamber (epidermal side down) to allow time-lapse confocal imaging.

**Figure 1:**
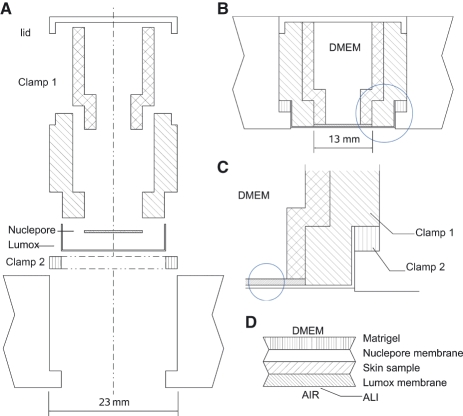
An ex vivo culture system for embryonic skin. (A) An expanded schematic of the components of the culture chamber. An embryonic skin sample (E14.5) is mounted epidermal side down between an 8.0 μm Nuclepore membrane (Whatman) and a Lumox (Greiner Bio-One GmbH) gas permeable membrane. (B) Schematic of the assembled chamber, several chambers can be mounted in a single block to allow multiple parallel experiments. (C) Detail of the area circled in (B), clamp 1 fixes the Lumox membrane tightly in place while clamp 2 presses the Nuclepore membrane down on top of the skin sample, sandwiching it between the two membranes. (D) Detail of the area circled in (C) showing the skin sample sandwiched between the two membranes: a layer of reduced growth factor Matrigel is placed on top of the Nuclepore membrane and the chamber is filled with culture medium (DMEM containing 5% fetal calf serum, 50 μg/ml Kanamycin, 25 mM HEPES). The chamber is mounted on the stage of a Leica SP5 confocal microscope enclosed by an environmental chamber providing 5% CO_2_ (in air) and a constant temperature of 37°C. ALI, air liquid interface; DMEM, Dulbecco’s Modified Eagle Medium.

In this system, the combination of the Nuclepore membrane and Matrigel provides support for the dermal side of the tissue, whilst the gas-permeable Lumox membrane allows an ALI to be maintained at the epidermal side, as well as providing a surface amenable to confocal imaging. The culture is fed from above by the diffusion of culture medium through the Matrigel and Nuclepore membrane. It should be noted that, whilst we achieved better results using a specially designed chamber to immobilise the sample, skin can be cultured in a similar configuration using 35 mm Lumox dishes (Sigma-Aldrich). In this case the skin sample is sandwiched between the base of the Lumox dish and a Nuclepore filter, ‘glued’ down using matrigel and the dish is then filled with culture medium. Figure 2(A) shows a typical field of cells from an E14.5 embryonic skin sample captured by confocal microscopy. In order to produce time-lapse series, images (single Z-planes) were captured every 7 min for up to 34 h in culture. Despite this relatively high and frequent laser exposure, skin cultures survived well and YFP expression was maintained throughout the culture period. Consequently, we were able to produce time-lapse movies of melanoblasts migrating throughout the embryonic epidermis. Video Clip S1 shows such a time-lapse experiment, in this example the culture was maintained for 8 h and images were captured every 7 min. Highly-motile melanoblasts exhibit a characteristic spindle shape and are seen to migrate apparently randomly throughout the tissue sample. Periodically cells are seen to stop migrating and round up before dividing to produce two motile daughter cells.

**Figure 2:**
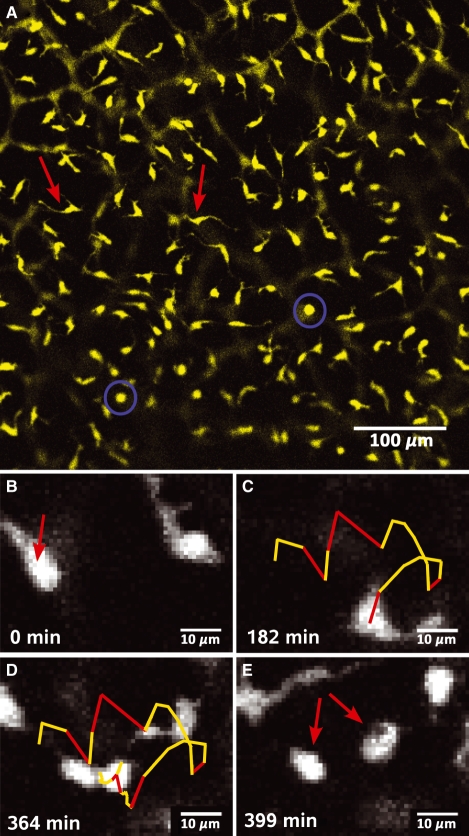
Live imaging of migrating melanoblasts in embryonic skin culture. Because the skin sample is flat and at E14.5 the majority of melanoblasts are located in the epidermis a single confocal Z-section can be used to capture a field of migrating cells. (A) A single image from a time-lapse series of migrating melanoblasts. Cells that are actively migrating exhibit a characteristic spindle-like shape (red arrows in A), while dividing cells appear rounded (blue circles in A). (B–E) Automated tracking of an individual melanoblast from the same time series using the ‘Particle detector and tracker’ plugin for ImageJ. The melanoblast in question (red arrow in B) migrates on a circular trajectory (C, D) for the first 182 min of the time series. It then slows virtually to a stop and undergoes a cell division. The melanoblast migrates 145.72 μm in 357 min at an average speed of 0.4 μm/min.

To demonstrate the importance of the ALI we disrupted it by placing an impermeable glass cover slip between the skin sample and the Lumox membrane. The resulting culture had approximately 50% of its surface area in contact with the Lumox membrane and the other 50% in contact with the cover slip. We imaged the culture so that half of the field of view was taken up by skin in contact with the Lumox membrane and the other half was skin in contact with the glass cover slip. Whilst melanoblasts cultured on a Lumox membrane remain migratory those cultured on a cover slip do not migrate and by 12-h the majority have died (see Video Clip S2). To study the kinetics of melanoblast migration we used the freeware image analysis software package ImageJ (http://rsb.info.nih.gov/ij/). The ‘Particle Tracker’ plugin is an automated Image J Plugin for multiple particle detection and tracking from digital videos that implements the algorithm described in Sbalzarini and Koumoutsakos (2005). This software allows the identification and tracking of individual cells and the plotting of their trajectories as well as providing the raw data to make calculations of speed and distance. An example is shown in Video Clip S3 and Figure 2(B, D).

In summary, we describe for the first time a method for the culture of embryonic skin in a manner that allows live cell imaging of melanoblast migration. The technique enables the study of melanoblast dynamics in an ex vivo environment allowing time-lapse imaging of their interactions with one another and the developing hair follicle for the first time. Preliminary studies suggest that melanoblast migration between E14.5 and E15.5 may be random. Cells tend to switch between migrating in straight and circular trajectories and do not seem to exhibit any directional movement towards developing follicles at this age. Cells pause before undergoing cell divisions and their daughter cells appear to migrate in opposite directions after the division. Initial calculations suggest that cells migrate at speeds of around 0.5 μm/min (n = 4 trajectories), but this includes periods of minimal movement as cells pause to divide, so the actual speed may be higher. In later stages of culture (E14.5 + 34 h) cells appear to aggregate within the developing hair follicle. We anticipate that this technique will be a powerful tool in the investigation of the developmental mechanisms that control melanoblast specification, migration, survival and localisation.

## Supporting information

**Video Clip S1.** Live imaging of YFP-labelled melanoblasts in embryonic skin. The system allows the resolution of individual cell shapes including the characteristic dendrites of migrating cells. Cells that are about to divide stop migrating before the cell division.

**Video Clip S2.** To demonstrate the importance of using a Lumox membrane to maintain a healthy culture system we placed a cover slip across part of the membrane in order to disrupt the ALI. We then imaged the sample with the edge between the two environments running down the middle of the field of view, the two cell populations have been pseudo-coloured red and green. Even in the very early stages of the time-lapse series, when the skin is presumably still (relatively) healthy, melanoblasts are immobile when cultured next to the cover slip (RED cells), whereas when cultured on a Lumox membrane (GREEN cells) they were able to migrate freely. After around 9 h in culture the cover slip inhibited cells are visibly beginning to die and non-appear to survive as far as 34 h. However the Lumox treated cells are still migrating and beginning to interact with the developing follicles at this stage (a developing follicle is circled in the last frame of the movie).

**Video Clip S3.** Automated cell tracking of a single melanoblast. The cell of interest migrates on a circular trajectory before rounding up and undergoing a cell division. The daughter cells migrate away from each other in opposite directions.
